# Quantitative and qualitative assessment of enamel surface alterations induced by three different interproximal reduction systems: A comparative *in vitro* study

**DOI:** 10.6026/973206300221203

**Published:** 2026-02-28

**Authors:** Karthick Shetty, Hemlata Ashok Jadhav, Aparna Khamatkar, Rakesh Avadesh Singh, Pranita Jadhav

**Affiliations:** 1 Department of Orthodontics and Dentofacial Orthopaedics, D.Y. Patil Deemed to be University School of Dentistry, Nerul, Navi Mumbai, India

**Keywords:** Interproximal enamel reduction (IPR), enamel surface roughness, scanning electron microscopy, orthodontics, enamel abrasion

## Abstract

Interproximal enamel reduction (IPR) is commonly used in orthodontics to address tooth-size discrepancies, but enamel removal is irreversible and may compromise surface integrity. Therefore, it is of interest to evaluate the quantitative and qualitative enamel surface alterations induced by three IPR systems: mechanical oscillating strips, diamond burs, and manual coarse strips. Thirty extracted human premolars were randomly assigned to three groups and subjected to IPR using different methods. Enamel thickness and surface roughness (Ra) were measured before and after IPR and surface morphology was analyzed using scanning electron microscopy. This study advances knowledge by providing a comparative analysis of IPR systems, aiding clinicians in selecting the most effective and biologically conservative technique for orthodontic treatment.

## Background:

Interproximal enamel reduction (IPR) is a routinely employed orthodontic procedure for managing tooth-size discrepancies and mild-to- moderate dental crowding through controlled removal of proximal enamel surfaces. Since its initial description by Sheridan in 1985 as air- rotor stripping, the technique has undergone substantial refinement, transitioning from a space-gaining alternative to extractions into a biologically oriented adjunct to contemporary orthodontic treatment planning [1]. Its relevance has further increased with the growing emphasis on non-extraction protocols and the widespread adoption of clear aligner therapy [2]. Compared with extraction-based approaches, IPR offers several clinical advantages, including conservation of the natural dentition, preservation of arch form, reduction in overall treatment duration, and improved post-treatment stability [3]. The procedure is also commonly indicated for correction of Bolton discrepancies, optimization of interproximal contacts, and reduction of open gingival embrasures or black triangles, particularly in adult orthodontic patients [4]. Nevertheless, IPR entails irreversible enamel removal, and inappropriate case selection or inadequate control during the procedure may adversely affect tooth structure and long-term dental health. Dental enamel represents the most highly mineralized tissue in the human body, composed predominantly of hydroxyapatite crystals arranged in a complex prismatic configuration [5].

Owing to its non-regenerative nature, preservation of enamel integrity is a central tenet of minimally invasive dentistry. Alterations in enamel thickness and surface morphology following IPR therefore assume considerable biological significance. Surface roughness, commonly quantified as arithmetic mean roughness (Ra), plays a decisive role in bacterial adhesion, plaque accumulation, and susceptibility to demineralization [6]. Increased surface irregularities facilitate retention of cariogenic microorganisms, thereby heightening the risk of enamel decalcification, especially in orthodontic patients who frequently experience challenges in maintaining optimal oral hygiene [7]. Beyond biological considerations, modifications in enamel surface topography may influence optical properties such as light reflection and translucency, potentially compromising esthetics and predisposing the surface to extrinsic staining [8]. The extent of enamel removal and the quality of the residual enamel surface are critical for the long-term success of IPR procedures. Various instruments, including manual abrasive strips and mechanical systems such as oscillating strips and diamond burs, differ in their impact on enamel characteristics [9]. Mechanical oscillating strips provide controlled enamel reduction with smoother surfaces, while diamond burs may cause excessive roughness if not properly polished. Despite the growing interest in the biological effects of IPR, comprehensive studies comparing enamel loss and surface morphology across systems are limited [10]. Therefore, it is of interest to assess the comparative biological effects of IPR systems on enamel structure.

## Materials and Methods:

This *in vitro* study was approved by the Institutional Review Board of D.Y. Patil Deemed to be University (DYPU/DENT/ EC/2024-XX). Thirty intact, non-carious human premolars extracted for orthodontic reasons from individuals aged 18-25 years were included, while teeth with restorations, structural defects, or enamel irregularities were excluded. Following extraction, specimens were ultrasonically cleaned and stored in 0.1% thymol solution at 4 °C to prevent dehydration and microbial contamination. Each tooth was embedded individually in self-curing acrylic resin within a custom mold, leaving only the mesial and distal proximal surfaces exposed. The occlusal surface was aligned parallel to the base to ensure procedural and measurement standardization. Specimens were randomly allocated using computer-generated randomization into three experimental groups (n = 10 each): Group A (mechanical oscillating strips), Group B (diamond burs), and Group C (manual coarse abrasive strips). Sample size estimation was performed using OpenEpi software (Version 3), based on pilot data obtained from five samples per group. Assuming an expected mean difference of 0.38 mm in enamel reduction, a standard deviation of 0.58, 80% statistical power, and a 95% confidence interval, a minimum of nine samples per group was required. To compensate for potential procedural or measurement variability, ten samples were included in each group. All IPR procedures were performed by a single calibrated operator with five years of orthodontic experience to minimize operator-related variability. Enamel reduction was standardized to 0.3 mm per proximal surface and verified using calibrated IPR gauges. In Group A, interproximal reduction was performed using a mechanical oscillating strip system in accordance with the manufacturer's recommended sequence, including graded enamel reduction followed by polishing. Group B underwent enamel reduction using fine-grit diamond burs in a high-speed handpiece with continuous water irrigation and light, controlled brushing strokes to minimize thermal effects. In Group C, enamel reduction was carried out manually using coarse diamond-coated metal strips applied with standardized reciprocating movements, with instruments replaced periodically to ensure consistent abrasiveness. Enamel thickness was measured at cervical, middle, and incisal/occlusal thirds of the proximal surface using a digital vernier caliper with 0.01 mm precision. Pre- and post-IPR measurements were recorded, and the mean of three readings was used for analysis. Surface roughness was assessed using a contact profilometer equipped with a diamond stylus. Three linear tracings were obtained perpendicular to the direction of enamel reduction, and arithmetic mean roughness (Ra, µm) values were recorded before and after IPR. For qualitative analysis, two representative samples from each group were processed for scanning electron microscopy. Specimens were dehydrated through graded ethanol concentrations, sputter-coated with gold, and examined at magnifications of 30x, 100x, and 300x to evaluate surface morphology. Statistical analysis was performed using IBM SPSS Statistics Version 25. Normality was assessed using the Shapiro-Wilk test. Intra-group comparisons were conducted using paired t-tests, while inter-group comparisons were analyzed using one-way ANOVA followed by Tukey's post-hoc test. Statistical significance was set at p < 0.05. Measurement reliability was assessed using intra-class correlation coefficients.

## Results:

All three interproximal enamel reduction (IPR) systems produced significant reductions in enamel thickness and increases in surface roughness (p < 0.001), with comparable baseline values among groups. Diamond burs showed the greatest mean enamel reduction (0.76 ± 0.14 mm), followed by manual coarse strips (0.53 ± 0.18 mm), while oscillating strips produced the least reduction (0.37 ± 0.24 mm). Surface roughness increased most with diamond burs (Δ Ra = 0.40 ± 0.10 µm) and coarse strips (0.33 ± 0.09 µm), and least with oscillating strips (0.14 ± 0.05 µm) (Table 1). Scanning electron microscopy revealed distinct enamel surface patterns among the IPR systems. Oscillating strips produced smooth, uniform abrasion with preserved prismatic architecture, whereas diamond burs caused deep, irregular grooves. Manual coarse strips showed heterogeneous abrasion patterns, while untreated enamel displayed intact prism structure and smooth interprismatic regions.

## Discussion:

The present study demonstrates that interproximal enamel reduction systems differ substantially in their biological impact on enamel thickness and surface integrity. Although all systems effectively created space, the extent and quality of enamel alteration were strongly influenced by instrument design and mode of action. Diamond burs produced the greatest enamel reduction, reflecting their high cutting efficiency. However, the magnitude of enamel loss observed raises biological concerns, as excessive reduction may approach recommended safety limits and compromise tooth structure, particularly when multiple proximal surfaces are treated [11]. In contrast, oscillating strip systems demonstrated superior control and predictability, which is especially relevant in digitally planned orthodontic treatments where precise space creation is critical. Manual coarse strips showed intermediate enamel reduction but greater variability, highlighting their technique-sensitive nature. Variations in applied pressure and angulation during manual IPR have been shown to result in inconsistent enamel removal and localized over-reduction [12]. Elevated surface roughness may predispose enamel to demineralization, particularly in orthodontic patients with compromised oral hygiene. In contrast, oscillating strips produced comparatively smoother surfaces, likely due to their integrated polishing sequence. The importance of polishing following IPR is well documented. Polishing duration and abrasive sequence have been identified as critical determinants of post-IPR surface quality, regardless of the primary enamel reduction method [13]. Accordingly, when burs or manual strips are used, polishing should be considered an essential step rather than an optional adjunct. SEM findings further supported the quantitative data. Oscillating strips produced fine, uniform abrasion patterns with minimal disruption of enamel prisms, suggesting controlled superficial enamel removal. Diamond burs generated deep grooves and microfractures consistent with aggressive rotational abrasion. Manual coarse strips produced irregular surface patterns, reflecting operator-dependent variability [14]. These findings align with contemporary studies reporting smoother enamel surfaces following complete oscillating strip sequences and rougher topography associated with rotary instrumentation. Additionally, thermal studies have demonstrated higher intrapulpal temperature increases with bur-based IPR, underscoring the need for adequate cooling and controlled application [15]. Overall, the results support a stratified approach to IPR instrument selection. Oscillating strips appear most suitable when enamel preservation and surface quality are priorities, while diamond burs should be reserved for cases requiring rapid enamel reduction and must be accompanied by meticulous polishing protocols.

## Conclusion:

Mechanical oscillating strips produced the most conservative enamel reduction, preserving surface integrity and minimizing morphological disruption. Diamond burs caused the greatest enamel loss and surface irregularity, emphasizing the need for careful finishing. Thus, we show the importance of selecting appropriate IPR systems to balance efficiency and enamel preservation in orthodontic treatment.

## Figures and Tables

**Table 1:** Changes in enamel thickness and surface roughness following interproximal enamel reduction

**Group**	**Pre-IPR Enamel Thickness (mm)**	**Post-IPR Enamel Thickness (mm)**	**Mean Enamel Reduction (mm)**	**p-value**	**Pre-IPR Ra (µm)**	**Post-IPR Ra (µm)**	**Mean ΔRa (µm)**	**p-value**
A: Oscillating Strips	2.22 ± 0.18	1.85 ± 0.09	0.37 ± 0.24	0.001	0.69 ± 0.03	0.83 ± 0.03	0.14 ± 0.05	<0.001
B: Diamond Burs	2.10 ± 0.13	1.34 ± 0.09	0.76 ± 0.14	<0.001	0.73 ± 0.07	1.12 ± 0.08	0.40 ± 0.10	<0.001
C: Coarse Strips	2.18 ± 0.15	1.65 ± 0.09	0.53 ± 0.18	<0.001	0.68 ± 0.08	1.01 ± 0.07	0.33 ± 0.09	<0.001
